# Self-reported and clinical periodontal conditions in a group of Eastern European postpartum women

**DOI:** 10.1371/journal.pone.0237510

**Published:** 2020-08-18

**Authors:** Iulia C. Micu, Sorana D. Bolboacă, Gabriela V. Caracostea, Diana Gligor, Andreea Ciurea, Sofia Iozon, Andrada Soancă, Daniel Mureșan, Alexandra Roman

**Affiliations:** 1 Department of Periodontology, “Iuliu Haţieganu” University of Medicine and Pharmacy, Cluj-Napoca, Romania; 2 Department of Medical Informatics and Biostatistics, “Iuliu Hațieganu” University of Medicine and Pharmacy Cluj-Napoca, Romania; 3 Department of Obstetrics and Gynecology, “Iuliu Haţieganu” University of Medicine and Pharmacy, Cluj-Napoca, Romania; 4 Emergency County Clinical Hospital, Cluj-Napoca, Romania; Centre Hospitalier Regional Universitaire de Tours, FRANCE

## Abstract

Periodontitis is a highly prevalent condition leading to a continuous destruction of tooth-supporting tissues. It increases the risk for various systemic diseases and adverse pregnancy outcomes. Therefore, screening for periodontitis is important. Screening measures can range from self-reported symptoms to clinical full-mouth periodontal examination. The hypothesis of our study was that self-reported parameters and clinical definition perform equally well in identifying periodontitis patients. The aim of this study was to develop, validate its internal consistency, and evaluate a self-reported instrument against periodontal clinical evaluation for diagnosis of periodontitis in a group of postpartum women, as well as to describe their periodontal status and the risk factors associated with periodontal disease. A cross-sectional study on postpartum women was conducted in a tertiary university hospital, from April 2018 to March 2019. Sociodemographic and behavioral data, periodontal clinical parameters, and self-reported periodontal perception were collected. A 16-item questionnaire was developed to obtain information about perceived periodontal alterations and oral hygiene habits. The utility of the questionnaire was tested against a periodontal diagnosis based on a full-mouth periodontal examination. The questionnaire was applied in 215 postpartum women aged 29.16±5.54 years (mean age (y) ± standard deviation) having the following periodontal status: 16 individuals without periodontal disease (7.44%), 32 individuals with gingivitis (14.88%), 19 individuals with mild periodontitis (8.84%), 132 individuals with moderate periodontitis (61.39%), and 16 individuals with severe periodontitis (7.44%). A significant association was observed between oral hygiene score, smoking status, and periodontal conditions (p<0.05). A significant association between the self-reported items related to “gum swelling”, “halitosis”, “previous periodontal diagnosis” and “previous periodontal treatment” with clinical periodontitis have been identified (p<0.05). Using self-reported questionnaires for detection of periodontal disease was ineffective in our studied population, since self-reported parameters and clinical definition do not appear to perform equally in identifying periodontitis cases. Clinical periodontal examination remains the gold standard for screening. Periodontitis was frequent in our group and the severity was significantly associated with the oral hygiene score and smoking. These results underline the necessity for periodontal clinical examination during pregnancy.

## Introduction

Periodontitis is an infectious, inflammatory condition initiated by specific microorganisms leading to a continuous destructive process of tooth-supporting tissues. Periodontitis is highly prevalent, affecting approximately 47% of adults that represent 64 million people in the United States [1].

Periodontitis is considered a major global public health problem causing tooth loss, disability, masticatory and speech dysfunction, poor nutritional status, and reduced quality of life [2]. About 57 systemic conditions have been hypothesized to be linked with periodontitis through infectious-inflammatory pathways, covering nearly 2% of the diseases indexed in the Medical Subject Headings [3].

Maternal oral infections, such as acute gingival infections and periodontitis, may be independent contributors to poor pregnancy outcomes, including preterm birth, growth restriction, and preeclampsia [4–6]. These are serious events that cause death or disability in newborn infants and mothers worldwide [7].

Immunological and hormonal alterations, as well as shifts in the composition of subgingival pathogens during pregnancy, could play a role in the onset and further development of pregnancy gingivitis. Also, pregnancy is supposed to increase the risk of new-onset periodontal disease due to important systemic changes by increasing local gingival inflammation [8, 9]. Specific data regarding the prevalence of periodontal disease during pregnancy is scarce, and reported values range from 30% to 100% for gingivitis and from 5% to 100% for periodontitis [8, 9]. The wide ranges can be explained by the variety of case definitions used in different studies [10] as well as the variability of the groups. Pregnancy can also worsen the periodontal status of already preexisting periodontitis [9]. However, despite perceived changes in their oral health, pregnant women rarely consult their dental clinical practitioner except in cases of emergency when they are in pain [11].

Epidemiological studies are important for estimating disease prevalence, developing preventative methods, and creating control strategies [12]. Clinically based full-mouth evaluation is considered the gold standard for surveying periodontitis and determining its prevalence [13, 14] as well as for screening of periodontitis in the dental office [12, 14–16], but it is time-consuming and expensive [12, 14, 15]. However, due to the discrete symptoms intensity during the initial phases of development, periodontitis is hardly recognized by patients. An accessible, reliable, cost-effective, time-saving strategy to facilitate periodontal disease detection could be beneficial to guide the subjects toward specialized evaluation [7, 12, 15, 17, 18].

An alternative method to detect periodontitis associated symptoms that could target a larger population outside dental offices is self-reported instruments. Self-report is an efficient, more attainable and accepted means of assessing sociodemographic information, population characteristics, risk factors, behaviors, and diseases, but has rarely been used for periodontal disease screening [13, 14, 17]. Self-reported measures could facilitate the screening of periodontitis in a larger population [17] and eventually, the development of state and local public oral health [13, 17] or education programs [19]. Moreover, self-reported measures could facilitate ongoing studies to evaluate associations between periodontitis and other diseases and conditions [12, 17].

Sparse data concerning self-reported periodontal problems compared with clinical assessment are available [12, 13, 15, 18, 19]. To date, no self-reported screening attempts addressing periodontitis have been developed in Romania. It is believed that pregnant women are more vigilant about their own health in order to carry a healthy baby to term. As a result, they are more willing to identify any health-related problems. The hypothesis of our study was that self-reported parameters and clinical definition perform equally well in identifying periodontitis patients. Based on this assumption, our primary objective was to develop, validate its internal consistency, and evaluate a self-reported periodontitis instrument and compare it to periodontal clinical diagnosis in a group of postpartum women. The description of the periodontal status, along with the risk-related factors of this group based on clinical assessment, was the secondary objective.

## Materials and methods

### Study design and participants

A cross-sectional study of postpartum women who delivered a live infant prior to examination was conducted. The study was performed in a tertiary university hospital, Gynecology Clinic 1, Emergency County Clinical Hospital, Cluj-Napoca, after receiving ethical approval from the Hospital Ethics Committee (957/24.10.2017) and “Iuliu Hațieganu” University of Medicine and Pharmacy, Cluj-Napoca (9/12.01.2018). Written informed consent was obtained from each of the participants before they answered the written questionnaire and underwent the clinical periodontal examination. In obtaining informed consent and conducting the evaluations, the study adhered to principles outlined in the Declaration of Helsinki on experimentation involving human subjects.

All subjects completed the survey within the first 72 hours after delivery for general demographic data and behaviors, self-reported periodontal conditions, and oral hygiene habits. The periodontal examination in hospitalization conditions was carried out within the same day when the questionnaire was filled out. Data were collected from April 2018 to March 2019. Every ninth subject admitted at the Gynecology Clinic 1, Emergency County Clinical Hospital, Cluj-Napoca who met the inclusion criteria in this period was selected to participate in the study. If the subject refused to participate in the study, the next admitted patient was selected.

### Inclusion and exclusion criteria

Postpartum women were eligible for the study if they were aged ≥ 18 years, able to read and understand Romanian, and had delivered a live infant in our hospital. The exclusion criteria were: 1) women aged < 18 years 2) any systemic disease that could possibly influence the history of periodontitis (e.g. chronic hypertension, pregestational diabetes, chronic inflammatory diseases, etc.); 3) any medical condition requiring antibiotic prophylaxis for dental treatment or systemic antibiotic treatment within the last 3 months; 4) human immunodeficiency virus (HIV) infection.

### Sociodemographic data

Sociodemographic data were collected from medical records. The following items were collected: 1) education level (level 1 = up to gymnasium, level 2 = high-school, level 3 = university degree); 2) urban or rural domicile of origin; 3) ethnicity (Romanian, Hungarian, German, or Other); 4) average income per family member (≤250€, 251–600€, ≥ 600€); 5) smoking status (smoker, nonsmoker, or ex-smoker). The economic status was evaluated according to the national criteria for the definition of poverty, based on the monthly income per family member.

### Questionnaire-collected information

A 16-item questionnaire (Table 1, S1 Appendix) was used to obtain information about perceived periodontal alterations (9 items) and oral hygiene habits (7 items). The self-reported periodontitis symptoms section of the questionnaire contained 9 questions and was created by 3 native-speaking Romanian periodontologist seniors. As a template for the questionnaire, the periodontists began with a previously validated Center for Disease Control/American Academy of Periodontology (CDC/AAP) 8-item self-report tool [13, 17]. Four items were adapted from the questionnaire elaborated by Eke et al. [13] (I.3 Visible roots; I.5 Tooth mobility; I.8 Previous periodontal diagnosis; and I.9 Previous periodontal treatment). Five additional items that seemed relevant as signs of periodontitis (I.1 Bleeding gums; I.2 Gum swelling; I.4 Tooth migration; I.6 Tooth loss; and 1.7 Halitosis) were selected based on data provided by other publications on self-reported periodontitis survey questionnaires [15, 20–23]. A 3-grade answer scale (Yes, No, or I am not sure) was available for each question.

**Table 1:** Self-reported items of the questionnaire.

	No	Question (item)	Abbreviation
**Self-reported periodontitis**	**I.1**	Have you noticed your gums bleeding while tooth brushing or chewing?	Bleeding gums
	**I.2**	Do you think that your gums are swollen?	Gum Swelling
	**I.3**	Do you think you can see more of the teeth’s roots than in the past?	Visible roots
	**I.4**	Have you noticed that your teeth changed their position lately?	Tooth migration
	**I.5**	Have you noticed any tooth become loose (mobile) lately?	Tooth mobility
	**I.6**	Have you lost any teeth in recent years?	Tooth loss
	**I.7**	Have you noticed having bad breath?	Halitosis
	**I.8**	Have you ever been told by a dentist that you need treatment for gum disease or periodontitis?	Previous diagnosis
	**I.9**.	Have you ever had periodontal treatment for gum disease and periodontitis?	Previous treatment
**Self-reported oral hygiene habits**	**II.1**	How often do you brush your teeth?	Tooth brushing frequency
	**II.2**	How often do you change your toothbrush?	Toothbrush changing interval
	**II.3**	Besides tooth brushing, I also use mouthwashes	Mouthwash
	**II.4**	Besides tooth brushing, I also use dental floss	Dental Floss
	**II.5**	Besides tooth brushing, I also use interdental brushes	Interdental brush
	**II.6**	Did a healthcare giver (doctor, nurse, medical student) ever explain to you the correct tooth brushing technique?	Previous brushing instruction
	**II.7**	How often do you visit your dental practitioner (besides emergency appointments)?	Periodic dental check-up

A former validated questionnaire was readapted to collect oral hygiene behavioral data [24]. The oral hygiene part of the questionnaire contains information about toothbrushing frequency, the intervals of toothbrush changing, the type and use frequency of interdental auxiliary hygiene aids (e.g., mouthwash, dental floss, and interdental brushes), the previous professionally supervised oral hygiene, and the periodic dental check-ups [24].

Our English version of the 9-item self-report periodontitis questionnaire was translated into Romanian independently and blindly by 3 experienced periodontists as well as by a native-speaking certified translator. The obtained translations were compared and discussed, and the first version of the Romanian self-report questionnaire was finalized. As recommended by Beaton et al, a cultural adaptation through consensus on the meaning of the translation of the problematic items was made [20]. The clarity for non-specialists and the comprehensibility of the questionnaire was further assessed on a group of 20 consecutive patients undergoing dental care at the Periodontology Department of “Iuliu Hațieganu” University, Emergency County Clinical Hospital, Cluj-Napoca. This alternative group was selected due to the low adhesion previously observed in postpartum women with respect to participation in clinical studies [25]. After filling in the questionnaire and discussing with a senior expert some changes in the wording and refining in the presentation of the questions were performed. The final version of the Romanian self-report questionnaire was finalized (Table 1). The validation of the Romanian version of the questionnaire was performed against a periodontal diagnosis based on a full-mouth periodontal examination considered as the gold standard for predictive validity (CDC/AAP case definition) [26, 27].

### Periodontal examination and diagnosis

The postpartum women included in the study underwent a full-mouth periodontal examination with standard methodology and equipment. The oral health examination was done in natural light supplemented by a top-of-the-range front flashlight using a dental mirror and a 1-mm marking periodontal probe (UNC-15 periodontal probe, Hu-Friedy, Chicago, IL, United States). Six sites per each tooth were evaluated for probing depth (PD), gingival recession, and clinical attachment loss (CAL) according to standard clinical definitions [26]. All the teeth (excluding wisdom teeth) yielding 168 sites in a fully dentate individual were evaluated. All probing measurements were rounded down to the nearest millimeter. Bleeding on probing (BoP) was also performed, and a full-mouth BoP score (%) was calculated as the total number of sites with gingival bleeding on probing divided by the total number of sites per mouth (6 sites at each tooth), multiplied by 100 [28]. Oral hygiene was rated using the oral hygiene score (OHS). Scraping was performed in 3 sites at each tooth and calculated as a percentage [29]. The periodontal examination took 30 minutes on average.

Maternal postpartum periodontal status was defined initially as a 5-level categorical variable (health, gingivitis, mild -, moderate—and severe periodontitis). A 3-level periodontitis variable (severe, moderate, and mild periodontitis) was established according to CDC/AAP definitions based on measures of CAL and PD at interproximal sites [26, 27]. Severe periodontitis was defined as having at least 2 sites with ≥ 6 mm of CAL (not on the same tooth) and at least 1 interproximal site with ≥ 5 mm of PD. Moderate periodontitis was defined as 2 or more interproximal sites with ≥ 4 mm of CAL (not on the same tooth) or 2 or more interproximal sites with ≥ 5 mm of PD, also not on the same tooth. Mild periodontitis was defined as at least 2 interproximal sites with ≥ 3 mm of CAL and at least 2 interproximal sites with ≥ 4 mm of PD (not on the same tooth) or 1 site with ≥ 5 mm. Gingivitis and periodontal health completed the 5-level categorical variable. Patients with gingivitis did not meet the preceding definitions but had a BoP score ≥ 10% [30]. The remaining women were considered clinical periodontal healthy (BoP < 10%) [30].

For the aforementioned periodontal conditions, OHS and missing teeth were reported as the median (first to third quartile) and residual roots as minimum to maximum range.

### Investigator training

Periodontal measurements and recordings were performed by 4 experienced investigators (I.C.M., A.S., A.C., S.I.) whose measurements were calibrated in the presence of a senior periodontist. All investigators attended 2 training and calibration meetings; they received oral and written instructions on the development of the study, measurement techniques, and data compilation sheets and were given their precise role and responsibilities in the study. To evaluate intra-examiner and inter-examiner reproducibility, 4 subjects not involved in the study but who met the enrollment criteria were evaluated on 2 occasions, 24 hours apart. The intra-class correlation coefficients, used as a measure of intra-examiner and inter-examiner reliability, were 0.95 and 0.94, respectively.

### Data analysis

Experimental data were analyzed with the Statistica program version 13 (StatSoft, Tulsa, OK, United States) at a significance level of 5%. The internal consistency of the proposed tool in the identification of periodontitis was tested with Cronbach alpha after splitting the sample into 1) odd- vs. even-numbered items and 2) 30 randomly determined split-half designs with the correlation of the subtotals for 8 and 7 of the scoring items. Excellent internal consistency is present for Cronbach alpha ≥ 0.9, good for values ≥ 0.8 and < 0.90, acceptable for values ≥ 0.7 and < 0.8, and questionable for values ≥ 0.6 and < 0.7 [31].

The number and the associated percentage were used to report the qualitative data. Chi-square test or Fisher exact test was used to test the association in contingency tables. Quantitative data were reported as median and the value of the first to third quartile (provided in round brackets) and compared using the Kruskal-Wallis test at a significance level of 0.01 (adjusted to the number of groups) due to the number of patients in each subgroup (health, gingivitis, mild periodontitis, moderate periodontitis, and respective severe periodontitis). The variability of OHS among groups was plotted using a box and whisker graph, and comparison between the 2 groups was tested with the Mann-Whitney *U* test. The ability of individual questions to serve as predictors for periodontitis was tested and reported following the guidelines presented by Bolboacă [32].

## Results

### Reliability analysis

The final version of the questionnaire was applied in a group of 215 postpartum women. Cronbach alpha applied to the history of dental health problems (9 items) and to oral hygiene behavior (6 items, the frequency of visiting the dentist not considered) had a value of 0.729 and a standardized value of 0.722. The estimated reliability of the scale for adding 8 new questions to the survey is 0.80, under the assumption of a similar inter-correlation between new items. A split-half reliability coefficient equal to 0.729 was obtained for the first strategy of internal reliability analysis (even vs. odd item numbers). The average of the split-half reliability coefficient calculated on 30 random half splits was 0.734, showing the extent of equal contribution of the items to the identification of the periodontitis.

### Descriptive analysis—General characteristics and periodontal status of the study group

The eligible population was comprised of 244 women, but 29 refused to participate in the study leading to a participation rate of 88%. Two-hundred fifteen postpartum women with a mean age (y) ± SD (29.16 ± 5.54) fulfilled the inclusion criteria, completed the 2-part questionnaire, and received a full-mouth periodontal examination.

According to the CDC/AAP epidemiological definition of periodontitis, the sample included 16 individuals without periodontal disease (clinical periodontal healthy) (7.44%), 32 individuals with gingivitis (14.88%), 19 individuals with mild periodontitis (8.84%), 132 individuals with moderate periodontitis (61.39%), and 16 individuals with severe periodontitis (7.44%).

Descriptive sociodemographic data in different periodontal conditions are presented in Table 2. A significant association was observed between smoking status (yes, no, or ex-smoker) and periodontal conditions (healthy, gingivitis, or periodontitis) with a significantly higher percentage of smokers with moderate or severe periodontitis (χ^2^ test, 10.5; p value = 0.0330).

**Table 2:** Sociodemographic characteristics of the group.

Item	Level	Total (n = 215)	Health (n = 16)	Gingivitis (n = 32)	Periodontitis			Stat. (P-value)
					Mild (n = 19)	Moderate (n = 132)	Severe (n = 16)	
**Education**	Level 1	32 (14.9)	3 (18.8)	5 (15.6)	4 (21.1)	18 (13.6)	2 (12.5)	n.a. (0.0506)
	Level 2	70 (32.6)	1 (6.3)	6 (18.8)	5 (26.3)	49 (37.1)	9 (56.3)	
	Level 3	113 (52.6)	12 (75.0)	21 (65.6)	10 (52.6)	65 (49.2)	5 (31.1)	
**Residence**	Urban	134 (62.3)	10 (62.5)	20 (62.5)	13 (68.4)	82 (62.1)	9 (56.3)	0.55 (0.9717)
	Rural	81 (37.7)	6 (37.5)	12 (37.5)	6 (31.6)	50 (37.9)	7 (43.8)	
**Ethnicity**	Romanian	186 (85.1)	14 (87.5)	27 (84.4)	15 (78.9)	115 (87.1)	12 (75.0)	n.a. (0.1668)
	Hungarian	19 (8.8)	0 (0.0)	4 (12.5)	2 (10.5)	11 (8.3)	2 (12.5)	
	German	12 (5.6)	2 (12.5)	0 (0.0)	2 (10.5)	6 (4.5)	2 (12.5)	
	Other	1 (0.5)	0 (0.0)	1 (3.1)	0 (0.0)	0 (0.0)	0 (0.0)	
**Income**	≤ 250€	58 (27.0)	3 (18.8)	7 (21.9)	10 (52.6)	32 (24.2)	6 (37.5)	n.a. (0.0864)
	251–600€	64 (29.8)	6 (37.5)	11 (34.4)	2 (10.5)	44 (33.3)	1 (6.3)	
	≥ 600€	93 (43.3)	7 (43.8)	14 (43.8)	7 (36.8)	56 (42.4)	9 (56.3)	
Smoking*	Smoker	51 (23.8)	2 (2.5)	3 (9.4)	3 (16.7)	38 (28.8)	5 (31.3)	n.a. (0.0581)
	Non-smoker	129 (60.3)	13 (81.3)	24 (75.0)	11 (61.1)	74 (56.1)	7 (43.9)	
	Ex-smoker	34 (15.9)	0 (0.0)	5 (15.6)	5 (27.8)	20 (15.2)	4 (25.0)	

The values in the table represent the number of cases and the associated percent provided in round brackets.

Level 1 = up to gymnasium; Level 2 = high-school; Level 3 = university education;

*Indicates n = 214.

Stat. (P-value): whenever n.a. the Fisher exact test was applied

The differences regarding the oral hygiene score, missing teeth, and residual roots according to the group are shown in Table 3.

**Table 3:** Oral hygiene score, missing teeth and residual roots by group.

Item	Health (n = 16)	Gingivitis (n = 32)	Periodontitis			Stat. (P-value)
			Mild (n = 19)	Moderate (n = 132)	Severe (n = 16)	
**OHS, %**	28.4 (17.4 to 44.5)	48.4 (35.2 to 63.4)	39.1 (24.3 to 67.8)	70.1 (38.7 to 89.5)	93.5 (89.2 to 98.9)	36.84 (<0.0001)
**Missing teeth**	4 (3 to 7)	5 (4 to 7)	6 (2 to 8)	4 (3 to 6)	6 (3 to 7)	1.57 (0.8134)
**Residual roots**	0 to 0	0 to 4	0 to 6	0 to 20	0 to 16	14.42 (0.0061)

OHS, oral hygiene score; Stat. = the statistics of the Kruskal-Wallis test and associated significance;

The values are expressed as median (first to third quartile) for OHS, and missing teeth; as minimum to maximum for residual roots

**Fig 1:**
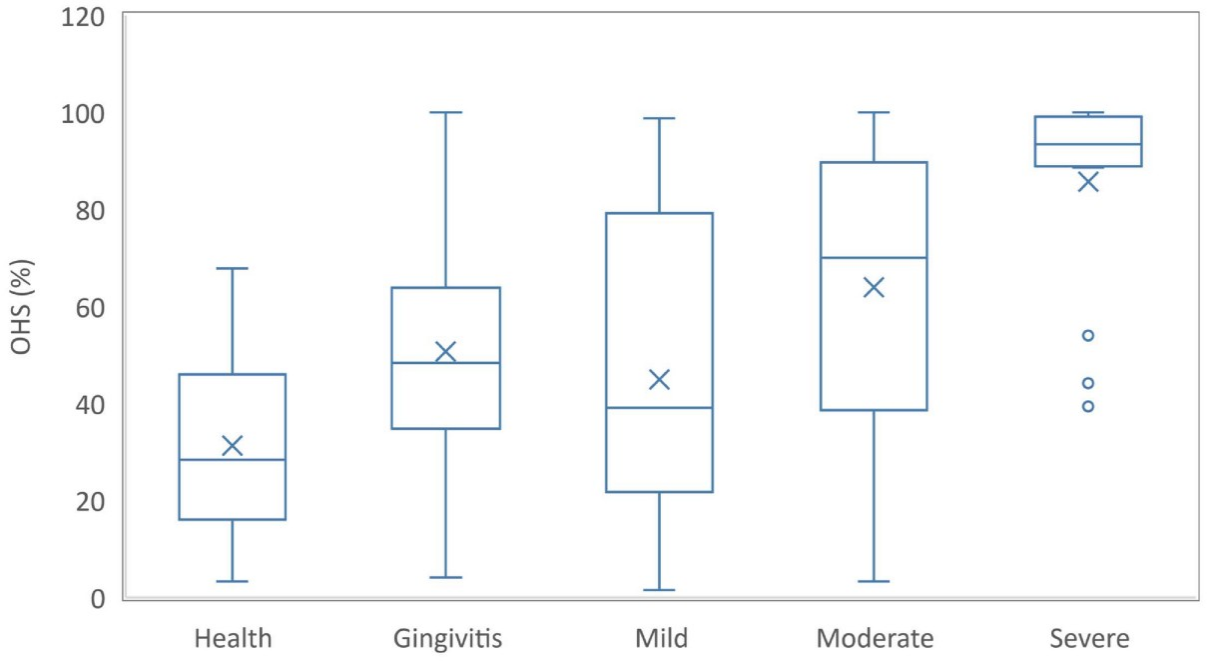
Oral Hygiene Score (OHS) distribution according to the group.

Significant differences were also observed regarding the oral hygiene parameter OHS (Fig 1). The p values associated to the OHS comparisons were as follows: 0.0087 for health vs. gingivitis; 0.0001 for health vs. moderate periodontitis; <0.0001 for health vs. severe periodontitis; 0.0203 for gingivitis vs. moderate periodontitis; 0.0001 for gingivitis vs. severe periodontitis; 0.0156 for mild periodontitis vs. moderate periodontitis; 0.0003 for mild periodontitis vs. severe periodontitis; and 0.001 for moderate vs. severe periodontitis.

Descriptive analysis related to self-reported periodontitis signs in relation to clinical diagnosis is presented in Table 4. The values are expressed as number and percentage according to the group (by column).

**Table 4:** Self-reported periodontitis signs in relation to clinical diagnosis.

Sign (Item)	Level	Total	Health (n = 16)	Gingivitis (n = 32)	Periodontitis			P-value
					Mild (n = 19)	Moderate (n = 132)	Severe (n = 16)	
**I.1. Bleeding gums**	Yes	56 (26.0)	4 (25.0)	5 (15.6)	11 (57.9)	32 (24.2)	4 (25.0)	0.1299
	No	77 (35.8)	8 (50.0)	12 (37.5)	4 (21.1)	47 (35.6)	6 (37.5)	
	I’m not sure	82 (38.1)	4 (25.0)	15 (46.9)	4 (21.1)	53 (40.2)	6 (37.5)	
**I.2 Gum swelling**	Yes	60 (27.9)	4 (25.0)	4 (12.5)	8 (42.1)	37 (28.0)	7 (43.8)	0.3210
	No	109 (50.7)	7 (43.8)	21 (65.6)	8 (42.1)	66 (50.0)	7 (43.8)	
	I’m not sure	46 (21.4)	5 (31.3)	7 (21.9)	3 (15.8)	29 (22.0)	2 (12.5)	
**I.3 Visible roots**	Yes	32 (14.9)	2 (12.5)	3 (9.4)	4 (21.1)	20 (15.2)	3 (18.8)	0.2695
	No	118 (54.9)	11 (68.8)	21 (65.6)	5 (26.3)	72 (54.5)	9 (56.3)	
	I’m not sure	65 (30.2)	3 (18.8)	8 (25.0)	10 (52.6)	40 (30.3)	4 (25.0)	
**I.4 Tooth migration**	Yes	34 (15.8)	2 (12.5)	7 (21.9)	5 (26.3)	16 (12.1)	4 (25.0)	0.3601
	No	143 (66.5)	9 (56.3)	21 (65.6)	10 (52.6)	94 (71.2)	9 (56.3)	
	I’m not sure	38 (17.7)	5 (31.3)	4 (12.5)	4 (21.1)	22 (16.7)	3 (18.8)	
**I.5 Tooth mobility**	Yes	24 (11.2)	0 (0.0)	3 (9.4)	1 (5.3)	17 (12.9)	3(18.8)	0.1507
	No	166 (77.2)	11 (68.8)	27 (84.4)	14 (73.7)	103 (78.0)	11 (68.8)	
	I’m not sure	25 (11.6)	5 (31.3)	2 (6.3)	4 (21.1)	12 (9.1)	2 (12.5)	
**I.6 Tooth loss**	Yes	36 (16.7)	3 (18.8)	3 (9.4)	5 (26.3)	20 (15.2)	5 (31.3)	0.1468
	No	167 (77.7)	10 (62.5)	28 (87.5)	13 (68.4)	106 (80.3)	10 (62.5)	
	I’m not sure	12 (5.6)	3 (18.8)	1 (3.1)	1 (5.3)	6 (4.5)	1 (6.3)	
**I.7 Halitosis**	Yes	45 (20.9)	2 (12.5)	3 (9.4)	7 (36.8)	26 (19.7)	7 (43.8)	0.1507
	No	103 (47.9)	7 (43.8)	16 (50.0)	8 (42.1)	66 (50.0)	6 (37.5)	
	I’m not sure	67 (31.2)	7 (43.8)	13 (40.6)	4 (21.0)	40 (30.3)	3 (18.8)	
**I.8 Previous periodontal diagnosis**	Yes	29 (13.5)	1 (6.3)	0 (0.0)	4 (21.1)	21 (15.9)	3 (18.8)	0.0174
	No	162 (75.3)	11 (68.8)	30 (93.8)	11 (57.9)	97 (73.5)	13 (81.3)	
	I’m not sure	24 (11.2)	4 (25.0)	2 (6.3)	4 (21.1)	14 (10.6)	0 (0.0)	
**I.9 Previous periodontal treatment**	Yes	19 (8.8)	0 (0.0)	1 (3.1)	2 (10.5)	15 (11.4)	1 (6.3)	0.5462
	No	178 (82.8)	13 (81.3)	28 (87.5)	16 (84.2)	108 (81.8)	13 (81.3)	
	I’m not sure	18 (8.4)	3 (18.8)	3 (9.4)	1 (5.3)	9 (6.8)	2 (12.5)	

The P-values represent the probability of the Fisher’s exact test

Self-reported oral hygiene behaviors are provided in Table 5. The values are expressed as number and percentage according to the group (by column).

**Table 5:** Self-reported oral hygiene behaviors.

Oral Hygiene Behavior (Item)	Level	Total	Health (n = 16)	Gingivitis (n = 32)	Periodontitis			P-value
					Mild (n = 19)	Moderate (n = 132)	Severe (n = 16)	
**II.1 Toothbrushing frequency**	≥ 2/d	169 (78.6)	13 (81.3)	24 (75.0)	15 (78.9)	104 (78.8)	13 (81.3)	0.9855
	≤ 1/d	46 (21.4)	2 (18.8)	8 (25.0)	4 (21.1)	28 (21.2)	3 (18.8)	
**II.2 Toothbrush changing interval**	At 3 mo	145 (67.4)	10 (62.5)	23 (71.9)	14 (73.7)	86 (65.2)	12 (75.0)	0.9021
	At 6 mo to 1y	61 (28.4)	5 (31.3)	8 (25.0)	4 (21.1)	41 (31.1)	3 (18.8)	
	> 1y	9 (4.2)	1 (6.3)	1 (3.1)	1 (5.3)	5 (3.8)	1 (6.3%)	
**II.3 Mouth wash**	≥ 1/d	75 (34.9)	3 (18.8)	11 (34.4)	7 (36.8)	49 (37.1)	5 (31.3)	0.7146
	< 1/d	140 (65.1)	13 (81.3)	21 (65.6)	12 (63.2)	83 (62.9)	11 (68.8)	
**II.4 Dental floss**	≥ 1/d	27 (12.6)	2 (12.5)	7 (21.9)	2 (10.5)	15 (11.4)	1 (6.3)	0.5719
	< 1/d	188 (87.4)	14 (87.5)	25 (78.1)	17 (89.5)	117 (88.6)	15 (93.8)	
**II.5 Interdental brush**	≥ 1/d	11 (5.1)	0 (0.0)	4 (12.5)	0 (0.0)	7 (5.3)	0 (0.0)	0.3130
	< 1/d	204 (94.9)	16 (100)	28 (87.5)	19 (100)	125 (94.7)	16 (100.0)	
**II.6 Previous brushing instruction**	Yes	120 (55.8)	9 (56.3)	20 (62.5)	9 (47.4)	72 (54.5)	10 (62.5)	0.8361
	No	95 (44.2)	7 (43.8)	12 (37.5)	10 (52.6)	60 (45.5)	6 (37.5)	
**II.7 Periodic dental check**	≤ 6 mo	55 (25.6)	2 (12.5)	11 (34.4)	4 (21.1)	36 (27.3)	2 (12.5)	0.5397
	annually	83 (38.6)	5 (31.3)	10 (31.3)	7 (36.8)	54 (40.9)	7 (43.8)	
	> annually	77 (35.8)	9 (56.3)	11 (34.4)	8 (42.1)	42 (31.8)	7 (43.8)	

The P-values represent the probability of the Fisher’s exact test

Only for moderate periodontitis, BoP significantly correlates with OHS (Spearman ρ = 0.6267; p<0.0001) and OHS with age (ρ = 0.1936; p = 0.0268). The association of BoP with OHS reaches the significance threshold for all possible answers on tooth loss (‘yes’, n = 45, ρ = 0.6104; p<0.0001; ‘no’, n = 103, ρ = 0.6452; p<0.0001; ‘I'm not sure’, n = 67, ρ = 0.5865; p<0.0001). However, the association between OHS and age reaches the significance only for participants who answer “I'm not sure” to the tooth loss (n = 67, ρ = 0.2846; p = 0.0206). No other significant association were identified when the subjects with different answers to oral hygiene and signs were investigated (p>0.05).

### Diagnostic performances

Four items of the survey, namely I.2, “Gum swelling”; I.7, “Halitosis”; I.8, “Previous periodontal diagnosis” and I.9, “Previous periodontal treatment” proved high specificity in evaluation of periodontitis (Table 6).

**Table 6:** The questions in the survey as predictors for periodontitis vs clinical periodontal diagnosis.

Item	I.2 Gum Swelling	I.7. Halitosis	I.8 Previous PO Diagnosis	I.9 Previous PO Treatment
True positive	52	40	28	18
True negative	40	43	47	47
False positive	8	5	1	1
False negative	115	127	139	149
Se, %	31.1 (24.1 to 38.2)	24.0 (17.5 to 30.4)	16.8 (11.1 to 22.4)	10.8 (6.1 to 15.5)
Sp, %	83.3 (72.8 to 93.9)	89.6 (80.9 to 98.2)	97.9 (93.9 to 100)	97.9 (93.9 to 100)
PPV, %	86.7 (78.1 to 95.3)	88.9 (79.7 to 98.1)	96.6 (89.9 to 100)	94.7 (87.7 to 100)
NPV, %	25.8 (18.9 to 32.7)	25.3 (18.8 to 31.8)	25.3 (19.0 to 31.5)	24.0 (18.0 to 30.0)
+LR	1.87 (0.95 to 3.66)	2.30 (0.96 to 5.50)	8.05 (1.12 to 57.63)	5.17 (0.71 to 37.77)
–LR	0.83 (0.70 to 0.97)	0.85 (0.75 to 0.97)	0.85 (0.79 to 0.92)	0.91 (0.85 to 0.97)
Accuracy, %	42.79 (36.18 to 49.40)	38.6 (32.10 to 45.1)	34.88 (28.51 to 41.25)	30.23 (24.09 to 36.37)
+CUI	0.27 (0.16 to 0.38)	0.21 (0.10 to 0.33)	0.16 (0.04 to 0.28)	0.10 (0.0 to 0.23)
–CUI	0.22 (0.14 to 0.29)	0.23 (0.16 to 0.30)	0.25 (0.18 to 0.31)	0.24 (0.17 to 0.30)

True positive indicate subjects with PO and a positive answer. True negative indicates subjects without PO and a negative answer. False positive indicates subjects without PO and a positive answer. False negative indicate subjects with PO and a negative answer.

+, positive;–, negative; CUI, clinical utility index; LR, likelihood ratio; NPV, negative predictive value; PO, periodontitis; PPV, positive predictive value; Se, sensitivity; Sp, specificity.

A few false-positive results were found indicating periodontal disease where there was no evidence of the disease on the clinical examination. On the contrary, many false-negative results became evident in cases where periodontal disease was found on examination, but the survey indicated otherwise.

## Discussion

Due to the ineffectiveness of periodontal epidemiological studies to detect periodontitis in the general population, this study aimed to set up a self-report questionnaire as an accessible and cost-effective tool to track periodontitis in large population groups that could be easily surveyed, such as with pregnant women. We chose this group of patients because of their presumably heightened physical awareness and interest in maintaining their health, for themselves and their future baby. The study also assessed the periodontal status and periodontal risk factors of postpartum women based on the examined clinical parameters.

An increased periodontitis frequency of 77.67% in this group of postpartum women was recorded when compared with the mean frequency of about 50% reported by the literature [1]. This finding may be due to the increased new-onset periodontitis induced by pregnancy [8, 9]. The proportion of women with severe periodontitis (7.44%) was lower than that in the general population (11%) [33], probably due to the lower mean age of our group.

From the evaluated periodontitis risk factors, smoking was significantly associated with periodontitis severity, which is to be expected considering the vast amount of data showing an increased prevalence, severity, and progression of periodontitis among smokers in comparison with nonsmokers or ex-smokers [34, 35]. Moreover, the outcomes of periodontal treatment are negatively influenced by smoking, but quitting smoking improves the clinical results [35]. The profound effect of smoking on periodontal tissues is related to the modifications of the oral microbiome (new bacteria acquisition through tobacco-infecting flora, increased biofilm formation, and tobacco-induced immunosuppression) [36], and local regenerative capabilities (harmful effects on gingival fibroblasts, mesenchymal stem cells, and bone metabolism) [37, 38].

As expected, the values of OHS significantly increased with the severity of periodontal involvement. Although it is a disease with a complex pathogenesis, periodontitis is primarily determined by the aberrant biofilm accumulations on tooth surfaces [39]. Periodontal inflammation is the natural consequence of dental microbial deposits [40] and it is even more increased during pregnancy [41].

The number of missing teeth is considered a surrogate indicator of periodontitis because it can be viewed as an index of lifetime accumulation of poor oral health [42]. No significant association between tooth loss and periodontitis was identified by the present study. Due to the relatively low mean age of our study group, the severity of the periodontal destruction was not as important to induce tooth loss.

The association analysis revealed significant results between self-report item I.2, “Gum swelling”; I.7, “Halitosis”; I.8, “Previous periodontal diagnosis”; and eventually I.9, “Previous periodontal treatment” and periodontitis (Table 6). Note that the answers for I.8 and I.9 may reflect a survey bias, namely a socially desirable answer. Furthermore, the access of participants to dental care could induce a bias since most of women were urban residents (Table 2) and dental medicine offices in urban areas are more common as compared to rural settings. The calculated sensitivity for these 4 items ranged between 10.8% to 31.1%, which is far less than the data of previously published self-report questionnaires for screening periodontitis using the same CDC/AAP case definition. They showed a sensitivity ranging from 44.3% to 85% [13–15, 43–45]. The specificity ranged from 83.3% to 97.9%, which is similar to the values varying from 58% to 92.8% reported by the previously mentioned studies. For the other items, no significant association with periodontal diagnosis was found. Based on our findings, this questionnaire could not accurately detect periodontitis cases.

We only referred to studies having the same CDC/AAP periodontitis case definition because changes in case definition affect the diagnostic accuracy of the self-report questionnaire and hamper the comparability of the results [25]. Carra et al, using the CDC/AAP case definition, communicated a self-report questionnaire validity only for severe periodontitis, although it is difficult to be missed either by patient or practitioner [15]. The present study used supplementary items other than those proposed by Eke et al [13] such as “Bleeding gums” (item I.1). “Bleeding gums”, especially bleeding on tooth brushing, is usually related to gingivitis, but also to periodontitis and it is highly exacerbated in periodontally affected pregnant women [41]. In our study “Bleeding gums” did not correlate with periodontitis possibly because its intensity was not proportional with the severity of the periodontal involvement. Gingival bleeding while brushing is the most frequently reported symptom, followed by gingival pain, gingival swelling, spontaneous gingival bleeding, and gingival redness [11]. Generally, the question on bleeding gums had low sensitivity with high specificity, that is, its absence is a good predictor of periodontal health [12]. However, one study showed that self-reported gingival bleeding was correlated with gingival bleeding on clinical examination [19]. Questions on bleeding gums need to be more detailed and a larger study is required to identify its validity [12].

Item I.2 “Gum swelling” was conceived in an effort to identify hyperplasic traits of inflammation associated with gingivitis and periodontitis in pregnancy, which would be identifiable by the patients. Our study showed some association of this item with clinical diagnosis (Table 6). Studies from different countries have shown that approximately 30% to 60% of the participants experience one or more self-report symptoms of gingival inflammation during pregnancy [11].

Items I.3 to I.6 were chosen so as to represent statements of some subjective periodontitis symptoms with an important effect on patients. For example, measures of severe diseases, such as tooth mobility and migration, as well as gingival recessions, may be easier for the patients to notice themselves. Subjects who face more disease might be more aware of their periodontal status [17] and, thus, we would have expected “Visible roots” (I.3), “Tooth migration” (I.4), “Tooth mobility” (I.5), and “Tooth loss” (I.6) to be valid measures of clinical periodontal status, especially of severe disease, which was not the case. Questions regarding tooth mobility and tooth migration had high specificity, but with different values of sensitivity [12].

The low sensitivity values recorded by our study for items I.3 to I.6 may be due to the fact that less than half of women with severe and moderate periodontitis reported the previously mentioned symptoms, that could be a characteristic of the investigated sample with a higher percentage of participants with urban residence and thus higher accessibility to dental medical services. It is possible that periodontitis was exacerbated by pregnancy [8, 9] causing important loss of attachment and deep pocket formation but without extensive bone loss leading to other obvious symptoms produced by extensive bone loss (e.g., visible roots, mobility). However, the accuracy and variability of responses to the questions are mediated by population factors, such as literacy, awareness, dental care habits, self-awareness of oral health, access to dental care, age, prevalence, and severity of periodontal disease [12, 13].

Halitosis is frequently related to periodontal disease, due to improper oral hygiene but most importantly due to the high proportions of subgingival Gram-negative, anaerobic bacteria which produce different volatile compounds as a result of their metabolic activity [39]. In our analysis, some association between item I.7 and clinical diagnosis was identified, probably because halitosis is a major complaint of most patients with periodontal disease (Table 6).

Item I.8, “Previous periodontal diagnosis” and I.9, “Previous periodontal treatment” have been previously reported to have a good validity [12, 13], but they did not fulfill the criteria of reliability to be used as a screening tool in our study (Table 6).

Although the association analysis of some of the items (I.2, I.7, I.8, and I.9) with the clinical periodontitis diagnosis (Table 6) was significant, these items are very poor both for case finding and screening. The important number of false-negatives related to items I.8 and I.9 implies that, although the clinical signs were present, postpartum women were incapable of detecting them. The low sensitivity and clinical utility index values prevent the validation of this self-report instrument. The AAP advises prenatal care visits as an opportunity for health care providers to educate women regarding behaviors and exposures that might affect their pregnancies and to offer proper periodontal examination and treatment for maintaining good oral health [46]. Periodontitis in pregnant women increases the risk of adverse pregnancy outcomes [5, 6, 47]. The benefits of intensifying oral hygiene measures during pregnancy should be extensively disseminated [11]. However, few obstetrician-gynecologists advise their patients to seek dental care, and many women decline dental services during this period [48]. No associations between oral hygiene habits and periodontitis were calculated.

One of the limitations of the present study is that the adaptation of some items from a questionnaire specifically developed for a population-based surveillance [13] could not be generalized across different groups. Furthermore, in the same context of questionnaire development, another limitation of our study is related to the validation of the proposed instrument, which was limited to internal consistency. However, the self-report questionnaire developed for the present study was not able to track any disease in our group, not even severe forms. However, the question identifying the hyperplasic inflammatory trait reached some level of significance, but it was singularly analyzed which could be considered as a limitation of the study. It was acknowledged that a single, self-reported item might not be accurate enough to discriminate between individuals with periodontal disease and those without it. Models combining both self-report measures and some demographic characteristics should provide more valid results [12, 15, 18]. Moreover, there is a great need to educate women in order to increase the perception about potential oral health changes during pregnancy as well as to raise the awareness regarding potential adverse pregnancy outcomes.

However, our self-report questionnaire could be further tested in the Romanian general population with a reliable stratification on age, gender and residence groups and a more accurate reflection of all stages of periodontal disease. Our team considers conducting such a study, after the completion of the research protocol and approval by the ethics committee. Furthermore, the utilization of the current definition of periodontal status [49] was also considered.

## Conclusions

Unfortunately, the results of the study prevent the validation of the self-report questionnaire as a screening tool for periodontitis in postpartum women. Self-reported parameters and clinical evaluation do not perform equally well in identifying periodontitis cases in this population group. Until the development of other tools, clinical evaluation remains the gold standard in screening for periodontitis. The frequency of periodontitis in our population group is high, and its severity was significantly associated with two preventable factors: oral hygiene score and smoking.

## Supporting information

S1 Appendix Questionnaire. (PDF)

S2 Appendix Raw data with meta-data. (XLSX)
