# Correction: Self-reported and clinical periodontal conditions in a group of Eastern European postpartum women

**DOI:** 10.1371/journal.pone.0245807

**Published:** 2021-01-14

**Authors:** Iulia C. Micu, Sorana D. Bolboacă, Gabriela V. Caracostea, Diana Grigor, Andreea Ciurea, Sofia Iozon, Andrada Soancă, Daniel Mureșan, Alexandra Roman

The fourth author's name is spelled incorrectly. The correct name is: Diana Grigor. The correct citation is: Micu IC, Bolboacă SD, Caracostea GV, Grigor D, Ciurea A, et al. (2020) Self-reported and clinical periodontal conditions in a group of Eastern European postpartum women. PLOS ONE 15(8): e0237510. https://doi.org/10.1371/journal.pone.0237510
