# Supplementary material for: Self-reported and clinical periodontal conditions in a group of Eastern European postpartum women
Source: PLoS One. 2020 Aug 18;15(8):e0237510. doi: 10.1371/journal.pone.0237510 (PMC7433868; doi:10.1371/journal.pone.0237510)
Supplement: S1 Appendix — (PDF) [file pone.0237510.s001.pdf]

Questionnaire

Personal information **Record number** **Date** **No.**

The two sections comprise of a series of questions regarding your periodontal health and personal dental hygiene habits. Check the boxes that best describe your situation. Please answer all questions accurate and honest.

Section I. Self-reported periodontal problems

I.1. Have you noticed your gums bleeding while tooth brushing or chewing?
- ❑ No
- ❑ I'm not sure
- ❑ Yes

I.2. Do you think that your gums are swollen?
- ❑ No
- ❑ I'm not sure
- ❑ Yes

I.3. Do you think you can see more of the teeth's roots than in the past?
- ❑ No
- ❑ I'm not sure
- ❑ Yes

I.4. Have you noticed that your teeth changed their position lately?
- ❑ No
- ❑ I'm not sure
- ❑ Yes

I.5. Have you noticed any tooth become loose (mobile) lately?
- ❑ No
- ❑ I'm not sure
- ❑ Yes

I.6. Have you lost any teeth in recent years?
- ❑ No
- ❑ I'm not sure
- ❑ Yes

I.7. Have you noticed having bad breath?
- ❑ No
- ❑ I'm not sure
- ❑ Yes

I.8. Have you ever been told by a dentist that you need treatment for gum disease or periodontitis?
- ❑ No
- ❑ I'm not sure
- ❑ Yes

I.9. Have you ever had periodontal treatment for gum disease and periodontitis?

- ❑ No
- ❑ I'm not sure
- ❑ Yes

Section II. Information regarding personal oral hygiene

II.1. How often do you brush your teeth?

- ❑ Twice a day or more than twice a day
- ❑ Once a day or less than once a day

II.2. How often do you change your toothbrush?

- ❑ Every 3 months
- ❑ Every 6 months to one year
- ❑ Less than once a year

II.3. Besides tooth brushing, I also use mouthwashes:

- ❑ Once a day or more than once a day
- ❑ Less than once a day

II.4. Besides tooth brushing, I also use dental floss:

- ❑ Once a day or more than once a day
- ❑ Less than once a day

II.5. Besides tooth brushing, I also use inter-dental brushes:

- ❑ Once a day or more than once a day
- ❑ Less than once a day

II.6. Did a healthcare giver (doctor, nurse, medical student) ever explain to you the correct tooth brushing technique?

- ❑ Yes
- ❑ No

II.7. How often do you visit your dental practitioner (*besides emergency appointments*)?

- ❑ At 6 months or more often
- ❑ Once a year
- ❑ Less than once a year
